# Relevance of the endoplasmic reticulum-mitochondria axis in cancer diagnosis and therapy

**DOI:** 10.1038/s12276-023-01137-3

**Published:** 2024-01-04

**Authors:** Garam An, Junho Park, Jisoo Song, Taeyeon Hong, Gwonhwa Song, Whasun Lim

**Affiliations:** 1 Institute of Animal Molecular Biotechnology, Department of Biotechnology, College of Life Sciences and Biotechnology, Korea University, Seoul, 02841 Republic of Korea; 2 Department of Biological Sciences, College of Science, Sungkyunkwan University, Suwon, 16419 Republic of Korea

**Keywords:** Mechanisms of disease, Stress signalling

## Abstract

Dynamic interactions between organelles are responsible for a variety of intercellular functions, and the endoplasmic reticulum (ER)–mitochondrial axis is recognized as a representative interorganelle system. Several studies have confirmed that most proteins in the physically tethered sites between the ER and mitochondria, called mitochondria-associated ER membranes (MAMs), are vital for intracellular physiology. MAM proteins are involved in the regulation of calcium homeostasis, lipid metabolism, and mitochondrial dynamics and are associated with processes related to intracellular stress conditions, such as oxidative stress and unfolded protein responses. Accumulating evidence has shown that, owing to their extensive involvement in cellular homeostasis, alterations in the ER–mitochondrial axis are one of the etiological factors of tumors. An in-depth understanding of MAM proteins and their impact on cell physiology, particularly in cancers, may help elucidate their potential as diagnostic and therapeutic targets for cancers. For example, the modulation of MAM proteins is utilized not only to target diverse intracellular signaling pathways within cancer cells but also to increase the sensitivity of cancer cells to anticancer reagents and regulate immune cell activities. Therefore, the current review summarizes and discusses recent advances in research on the functional roles of MAM proteins and their characteristics in cancers from a diagnostic perspective. Additionally, this review provides insights into diverse therapeutic strategies that target MAM proteins in various cancer types.

## MAM proteins: the hidden players in cancer diagnostics and therapy

Understanding the cooperation between organelles, especially the endoplasmic reticulum and mitochondria (the energy factories of cells), is vital to uncover how cellular functions are controlled. However, the exact function of the membranes linking these organelles, termed mitochondria-associated ER membranes (MAMs), in diseases like cancer is still uncertain. In this research, scientists found MAMs to play a vital role in cancer cell function and metabolism by regulating calcium signaling, lipid metabolism, autophagy, and mitochondrial fission. They discovered that changes in MAM proteins can result in inhibition of apoptosis and increased resistance to anticancer drugs. Therefore, therapies targeting MAMs may provide a new method for cancer treatment. The researchers also propose that MAM proteins could be used as diagnostic markers for specific cancers.

This summary was initially drafted using artificial intelligence, then revised and fact-checked by the author.

## Introduction

An understanding of the cooperation between organelles is crucial for revealing the mechanisms that modulate cellular functions and homeostasis. Among interorganellar networks, the connection between the endoplasmic reticulum (ER) and mitochondria has been extensively studied owing to its diverse functions and impact on the pathogenesis of multiple diseases. The concept of a functional unit comprising the ER and mitochondria was first proposed in 1950^1^. The adjacent membrane sites that physically tether the ER and mitochondria are called mitochondria-associated ER membranes (MAMs); technological advances in microscopy have enabled the elucidation of the physiological features of the tethering structures of the MAMs. The ER and mitochondria are separated by a 6–15 nm gap, and the average surface area percentage of mitochondria covered by MAMs was calculated to be 3–5% in mammalian cells^2^.

MAMs represent an etiological and therapeutic target in cardiovascular diseases^3^, neurodegenerative diseases^4^, metabolic disorders^5,6^, and cancers. In this review, we discuss the associations between alterations in MAM proteins and cancers and present recent advances in research on these associations. Additionally, we discuss the contribution of MAM proteins to tumorigenesis and cancer progression as well as their possible applications as diagnostic and therapeutic targets.

## Structure and functional role of ER–mitochondria contact sites

### Calcium regulation

Maintenance of Ca^2+^ homeostasis is one of the most important functions of MAMs, as the ER functions as the main regulator and storage organelle of calcium ions within living cells^7^. The resting levels of Ca^2+^ in mitochondria are similar to those in the cytosol; however, they can increase to 100 times the cytosolic levels under specific stimulation conditions^8^. A contributing factor to this drastic increase has been identified and subsequently confirmed by the Ca^2+^ microdomain hypothesis, which states that the outer membrane of mitochondria contains hotspots for Ca^2+^ shuttling from the ER^9–11^. As the affinity of the mitochondrial calcium uniporter (MCU) located in the inner mitochondrial membrane is dependent on the local Ca^2+^ concentration, these microdomains facilitate Ca^2+^ influx through the MCU^12,13^.

**Fig. 1:**
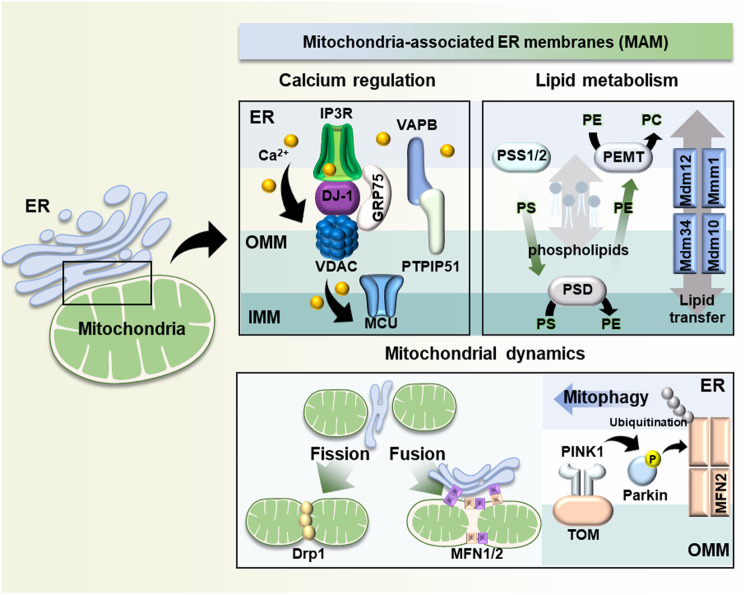
Representative roles of mitochondria-associated ER membranes (MAMs). The figure shows representative MAM proteins and their mechanisms of regulation of multiple cellular functions. (1) Calcium regulation is modulated mainly by the IP3R-GRP75-VDAC-MCU and VAPB-PTPIP51 complexes in MAMs. (2) Lipid synthesis and transfer are mediated by the enzymes PSS1/2, PSD, and PEMT and the Mdm12-Mmm1-Mdm34-Mdm10 complex. (3) Drp1 and MFN1/2 are involved in mitochondrial fission and fusion, respectively. The PINK1/Parkin pathway mediates mitophagy. ER endoplasmic reticulum, OMM outer mitochondrial membrane, IMM inner mitochondrial membrane. IP3R inositol 1,4,5-triphosphate receptor, GRP75 glucose-related regulated protein 75, VDAC voltage-dependent anion channel, MCU mitochondria calcium uniporter, VAPB vesicle-associated membrane protein B, PTPIP51 protein tyrosine phosphatase-interacting protein-51, PS phosphatidylserine, PE phosphatidylethanolamine, PC phosphatidylcholine, PSS1/2 phosphatidylserine synthase 1/2, PSD phosphatidylserine decarboxylase, PEMT phosphatidylethanolamine-*N*-methyltransferase, MFN1/2 mitofusin 1/2, PINK1 PTEN-induced putative kinase 1, TOM mitochondrial translocase of the outer membrane 70.

The translocation of Ca^2+^ in MAMs is mediated by several proteins. The inositol 1,4,5-trisphosphate (IP3) receptor (IP3R) is a representative Ca^2+^ channel located in the ER^14^ (Fig. 1). The opening of this receptor and subsequent Ca^2+^ transport occur when the binding site of each tetrameric subunit of IP3R is concatenated with IP3^15^. The IP3 binding affinity and Ca^2+^ influx activity of IP3R vary depending on its subtype^16^, phosphorylation^17^, and interactions with other regulatory proteins. Additionally, recent research has shown that the localization of mobile IP3R on MAMs is important for Ca^2+^ signaling between the ER and mitochondria^18^.

The canonical microdomain of the Ca^2+^ regulator in MAMs consists of IP3R located in the ER, voltage-dependent anion channel 1 (VDAC1) in the outer mitochondrial membrane (OMM), and glucose-regulated protein 75 (GRP75), which acts as a physical link between IP3R and VDAC1 and directly affects mitochondrial Ca^2+^ accumulation^19^. The formation of these complexes brings the ER and mitochondria into close proximity, resulting in the formation of microdomains with high Ca^2+^ levels^20,21^. Recent evidence has indicated the importance of another protein component within this microdomain. DJ-1 was recognized as the fourth component of the MAM complex through the observation that DJ-1 ablation induced IP3R3 aggregation, which prevented the tethering of the IP3R-GRP75-VDAC microdomain^22^.

In addition to the IP3R-GRP75-VDAC1 complex, the interaction of ER-integrated protein vesicle-associated membrane protein B (VAPB) with the OMM protein called protein tyrosine phosphatase-interacting protein-51 (PTPIP51) is also involved in Ca^2+^ regulation. Depletion of either VAPB or PTPIP51 leads to the disruption of MAMs and perturbation of Ca^2+^ transport^23^.

### Lipid metabolism

Because lipid synthesis is compartmentalized, lipids must be transferred between organelle compartments. Lipids shuttle between specific organelles through vesicle trafficking; however, lipid influx into mitochondria through vesicles is not possible even when lipids are needed^24^. Thus, several MAM proteins regulate the nonvesicular trafficking of lipids from the ER to mitochondria (Fig. 1). Phosphatidylserine synthase 1/2 (PSS1/2) is a representative synthetic enzyme that is enriched in MAMs and mediates phosphatidylserine (PS) synthesis^25^. Specifically, PSS1 and PSS2 convert phosphatidylcholine (PC) and phosphatidylethanolamine (PE), respectively, into PS. PE import relies on the conversion of transported PS in MAMs to PE by PS decarboxylase (PSD) in mitochondria rather than direct import^26^. Disruption of this process and the mitochondrial PE level impairs mitochondrial dynamics and bioenergetics^27,28^. Mitochondrial PE can be traced back to MAMs and is converted into PC by PE-*N*-methyltransferase^29^. This transfer system is the rate-limiting step in lipid biogenesis and further contributes to the maintenance of phospholipid homeostasis.

The complex consisting of Mdm10 and Mdm34 is located in the OMM, and Mmm1 in the ER and Mdm12 in the cytosol exhibit features of ER–mitochondria tethering proteins and phospholipid exchangers^30^. Mdm34, Mmm1, and Mdm12 physically interact with phospholipids via their synaptotagmin-like mitochondrial lipid-binding domains^31–33^. The direct binding of Mmm1 and Mdm12 forms a hydrophobic cavity that mediates the transport of glycerophospholipids except for PE^34^. However, as the depletion of this complex only exerts minor effects on the lipidome, more unknown lipid regulatory proteins and mechanisms may exist in MAMs^30^.

### Regulation of mitochondrial dynamics

Mitochondrial quality control is a defense mechanism against mitochondrial insult. In the early stages of quality control, translocation and recruitment of dynamin-related protein (DRP1) in mitochondria occurs in MAMs and facilitates mitochondrial fission^35^. In contrast, mitofusin 1 (MFN1), another MAM protein, forms puncta in the ER and facilitates mitochondrial fusion^36^. Physical tethering of the ER to mitochondria by MFN1/2 indicates the importance of MAMs as key sites for regulating mitochondrial dynamics^37,38^ (Fig. 1).

In addition to fission and fusion, self-degradation of mitochondria upon severe injury, a process called mitophagy, is also influenced by MAM proteins. The PTEN-induced putative kinase (PINK)/parkin pathway is the main signaling pathway for mitophagy. PINK is degraded by mitochondria-resident enzymes and further degraded in lysosomes under normal conditions; however, mitochondrial dysfunction leads to the formation of uncleaved PINK and its accumulation in the OMM through an interaction with TOM^39^. The accumulated PINK proteins recruit parkin, which induces mitophagy through its E3 ligase activity^39,40^. A recent study reported that PINK1/Parkin mediate MFN2 phosphorylation, resulting in the dissociation of the MFN2 complex via the p97-dependent pathway. This indicates a relationship between a decrease in ER–mitochondrial contact and mitophagy^41^. Additionally, assembly of the autophagosome marker ATG14 occurs in MAMs under starvation conditions, and disruption of the ER–mitochondria interaction inhibits ATG14 localization and autophagosome formation^42^.

## Interaction between the ER–mitochondrial axis and calcium homeostasis

Most ER proteins are involved in regulating Ca^2+^ homeostasis^43^. For instance, the sigma-1 receptor (Sig-1R), a MAM protein, is enriched in the ER vesicles involved in this process. In the resting state, Sig-1R binds to another chaperone in the ER, GRP78. However, this complex dissociates under ER stress conditions, including Ca^2+^ depletion. The dissociated Sig-1R then binds to IP3R, mediating its stabilization and Ca^2+^ influx^44^. PDZ domain-containing protein 8 (PDZD8) in the ER is another example of a Ca^2+^-regulating protein in MAMs. PDZD8 knockdown impairs ER–mitochondria tethering and further inhibits mitochondrial Ca^2+^ uptake in the MAMs of mammalian neurons^45^. Other proteins modulate calcium homeostasis in MAMs, as shown in Table 1.

**Table 1:** MAM proteins related to calcium homeostasis.

Name	Cells	Possible mechanisms
Sig-1R (sigma-1 receptor)	CHO cells	Mediates stabilizing IP3R and Ca^2+^ influx when dissociated from BIP
	Mouse, NRVM cells	Maintains the close proximity between IP3R2 and VDAC by interacting with IP3R2
PDZD8 (PDZ domain-containing protein 8)	Cortical pyramidal neurons, Drosophila	Mediates the tethering of EMCSs and mitochondrial uptake of Ca^2+^ through MCU
ALKBH5 (RNA demethylase alkb homolog 5)	143B, MG53, IMR90, and HEK293T cells	Modulates ER lipid raft associated 1 (ERLIN1)-IP_3_R Ca^2+^ signaling via hypermethylation of *ERLIN1* mRNA
FMRP (fragile X messenger ribonucleoprotein)	Drosophila, Mouse, U2OS, HEK293T, HeLa, and normal/patient derived fibroblasts	Regulates Ca^2+^ homeostasis by interacting with VDAC; loss of FMRP leads to excessive Ca^2+^ influx into mitochondria
TG2 (transglutaminase type 2)	CAD cells	Increases the number of IP3R-VDAC1 units through crosslinking amyloid beta
	HEK293 cells	Increases the number of EMCSs by interacting with GRP75 and increasing the formation of the IP_3_R-GRP75-VDAC1 complex
STING (stimulator of interferon response cGAMP interactor 1)	Human renal carcinoma cell lines	Interferes with interactions between VDAC1 and GRP75 by binding to VDAC1 in renal cancer cells
Pyk2 (proline-rich tyrosine kinase 2)	Mouse primary neuronal cells	Increases the number of EMCSs by regulating the protein expression levels of IP3R3 and VDAC1
S1T (sarcoendoplasmic reticulum Ca^2+^-ATPase 1)	HeLa cells	Leads to Ca^2+^ transport to mitochondria by increasing the number of EMCSs and inhibiting mitochondrial mobilization under ER stress conditions
TOM70 (mitochondrial translocase of the outer membrane 70)	Mouse, HeLa cells	Forms a cluster that contacts the ER and recruits IP3R3 to EMCSs via a physical interaction

As Ca^2+^ participates in diverse cellular processes, disrupted homeostasis and improper regulation of Ca^2+^ dynamics in MAMs can negatively affect cellular function. The influx of Ca^2+^ into mitochondria is essential for bioenergetics because several intramitochondrial enzymes associated with glycolysis and the tricarboxylic acid cycle are activated in a calcium-dependent manner^46^. The lack of constitutive Ca^2+^ influx through IP3 reduces the enzymatic activity of pyruvate dehydrogenase and thus the production of adenosine triphosphate (ATP), resulting in the activation of autophagy via the AMPK pathway^47^. Translocase of mitochondrial outer membrane 70 (TOM70) also affects constitutive Ca^2+^ shuttling by mediating the linkage between IP3R3 and VDAC, and the depletion of TOM70 results in impaired mitochondrial respiration^48^.

MAM proteins stimulate Ca^2+^-dependent apoptotic pathways. Ca^2+^ overload in the mitochondrial matrix increases mitochondrial permeability by opening mitochondrial permeability transition pores (mPTPs)^49^. One mechanism of permeability transition is Ca^2+^-inducible conformational alteration of F-ATP synthases that bind to and show activity toward mPTPs^50^. The opening of mPTPs disrupts the osmotic balance in mitochondria due to nonselective permeabilization, resulting in an influx of water that induces the release of caspase cofactors^51^. Furthermore, alterations in Ca^2+^ levels are closely associated with responses to multiple intracellular stresses, such as ER and oxidative stress.

## ER–mitochondria contacts modulate oxidative stress

Oxidative stress results from an imbalance between the production and accumulation of reactive oxygen species (ROS) in cells and is a hallmark of the ability to detoxify or repair reactive products^52^. ROS are produced primarily in mitochondria and play important roles in cell growth, differentiation, and death^53,54^. Although low levels of ROS play an essential role in intracellular signaling and pathogen defense, elevated levels can have detrimental effects on cells, such as decreasing the efficiency of mitochondrial respiration and inducing oncogenic stress^55^. Imbalances in ROS accumulation can contribute to the development and progression of several diseases, including cancer, metabolic disorders, diabetes, and cardiovascular diseases^56,57^.

**Fig. 2:**
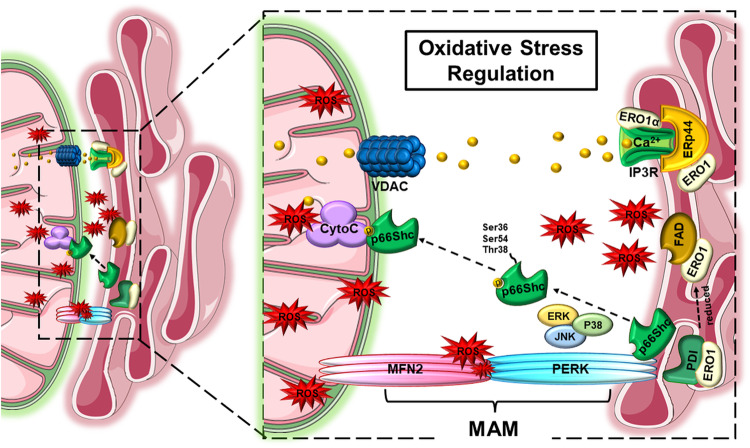
Mitochondria-associated ER membranes (MAMs) regulate oxidative stress. The figure shows the regulation of reactive oxygen species (ROS) production by several proteins present in the mitochondrial ER membrane. Reduced ERO1 generates ROS through an interaction with FAD. ERO1a, one of the ERO1 isoforms, regulates ROS production through an interaction with IP3R by releasing calcium ions into mitochondria, which induces chronic ER stress. Additionally, p66Shc modulates ROS production via phosphorylation at Ser36, Ser54, and Thr38 by ERK, JNK, and P38. Finally, MFN2 regulates ROS production in a manner dependent on its expression level through an interaction with PERK. ERO1 endoplasmic reticulum oxidoreductase 1, ERp44 endoplasmic reticulum protein 44, IP3R inositol 1,4,5-triphosphate receptor, GRP75 glucose-related regulated protein 75, VDAC voltage-dependent anion channel, CytoC cytochrome c, FAD flavin adenine dinucleotide, PERK protein kinase R-like endoplasmic reticulum kinase.

The ER and mitochondrial axes play essential roles in the detection of and response to stress conditions, including oxidative stress, and form interconnected networks^58^. Furthermore, the simultaneous induction of ER stress and overproduction of ROS in several diseases highlights the importance of this axis^59^. The roles of ROS-related MAM proteins, including endoplasmic reticulum oxidoreductase 1 (ERO1), Sig-1R, p66Shc, and MFN2, have been reported (Fig. 2).

ERO1 is located entirely on the MAMs close to the ER surface and is an essential factor in the ER oxidative folding mechanism through co-localization with protein disulfide isomerase (PDI)^60^. PDI catalyzes the formation of disulfide bonds in unfolded proteins during oxidative protein folding and is then converted to a reduced form^61^. Reduced PDI is subsequently oxidized by ERO1 to participate in the catalytic reaction cycle, where reduced ERO1 transfers electrons to an oxygen molecule via flavin adenine dinucleotide, releasing H_2_O_2_^60^. ERO1α, an ERO1 isoform, is overexpressed in various cancers, and its expression is increased by chronic ER stress, resulting in excessive H_2_O_2_ production and an increased ROS burden^62^. ERO1 also affects ROS production by regulating other MAM proteins. Under stress conditions, ERO1 oxidizes IP3R1 and induces detachment of the disulfide isomerase–like protein ERp44 from IP3R1^63^. ERp44 has an inhibitory effect on IP3R1^64^, leading to massive influx of Ca^2+^ through IP3R, which ultimately results in upregulated mitochondrial metabolism and excessive ROS production^65,66^.

Sig-1R regulates Ca^2+^ homeostasis and is involved in ROS-related signaling pathways. Although the ROS-regulatory mechanisms of Sig-1R are not fully understood, previous studies have shown that Sig-1R knockdown leads to ROS accumulation^67,68^. Furthermore, some Sig-1R agonists exhibit antioxidant activity under pathological conditions^69^.

p66Shc is located in MAMs, mitochondria, and the cytosol and tetramerizes in response to oxidative stress^70^. Under oxidative stress conditions, its Ser36 residue is phosphorylated by p38MAPK, ERK, and JNK1/2, and phosphorylation of other residues, namely, Ser54 and Thr386, occurs to prevent p66Shc degradation by ubiquitination^71–73^. Activated p66Shc translocates through MAMs into mitochondria, where it binds to cytochrome c to generate ROS and ultimately induce cell death^74^. The generation of ROS by activated p66Shc is supported by previous studies showing that both p66Shc knockout mice and cells exhibit reduced oxidative stress levels and a decreased incidence of diseases such as atherosclerosis^75,76^.

As previously described, both MFN1 and MFN2 are involved in the promotion of mitochondrial fusion. However, the fusion process, which relies primarily on MFN1 and MFN2, is speculated to have additional distinct functions^77^. The possible effects of MFN2 on ROS generation have been suggested to be due to other functions of MFN2. Munoz et al.^78^ reported the possible inhibitory effects of MFN2 on ROS production. MFN2 directly interacts with an ER stress branch, the pancreatic endoplasmic reticulum kinase (PERK) pathway, and inhibits ER stress pathways and ROS production. Other studies have shown that MFN2 overexpression activates the PERK/activating transcription factor 4 (ATF4) pathway and reduces ROS levels in cardiac fibroblasts^79^. However, a recent study showed that MFN2 facilitates the adaptation of macrophages to mitochondrial respiration and ROS generation in response to inflammatory stimuli^80^. Thus, further research is required to fully understand the different functions of MFN2 in different cell types and under specific stress conditions.

## Interaction between ER stress and the ER–mitochondria axis

Protein folding is the main function of the ER. Various conditions, such as disruption of Ca^2+^ homeostasis, inhibition of degradation of unfolded proteins due to proteasome blockade, and genetic mutations, can cause the accumulation of unfolded proteins^81^. Under these stress conditions, the unfolded protein response (UPR) is activated by three ER transmembrane proteins: activating transcription factor 6 (ATF6), inositol-requiring enzyme 1α (IRE1α), and PERK^82^. Under normal conditions, the ER chaperone GRP78/BiP binds to the ER lumen region of these transmembrane proteins and inhibits their activity. However, under stress conditions, GRP78 binds to misfolded proteins and induces the activation of these three transmembrane proteins^83^.

In the ATF6 pathway of the ER stress response, sensors mediate the UPR, and ATF6 translocates to the Golgi complex after GRP78 is released. ATF6 is first cleaved by site-1 protease, and one half remains at the NH_2_-terminus before being cleaved by site-2 protease^84^. Regarding the IRE pathway, GRP78 is normally bound to IRE1α or its homolog, IRE1p, and maintains its inactivation. When GRP78 dissociates from IRE1 in ER-stressed cells, IRE1 is phosphorylated and dimerizes^85^. Finally, activated PERK phosphorylates eIF2α and further increases the translation of selected mRNAs, including ATF4, which then promotes the expression of transcription factors, such as C/EBP homologous protein (CHOP), leading to growth arrest and DNA damage^86^. CHOP overexpression causes apoptosis by translocating B-cell lymphoma 2 (BCL2)-associated X (a proapoptotic protein) to mitochondria and decreasing the expression of BCL2 (an antiapoptotic protein)^87^.

The associations between MAM components and ER stress have been widely reported (Table 2), and some UPR-related proteins also function as MAM components. The interaction between PERK and MFN2 is a representative example of the UPR-related MAM pathway. Additionally, some MAM proteins are regulated by ER stress; for instance, Sig-1R is transcriptionally upregulated via the PERK/eIF2α/ATF4 pathway^88^, while another MAM protein, Rab32, is upregulated via the UPR pathway. Rab32 belongs to the Ras-like small GTPase family and is involved in mitochondrial fission via interaction with DRP1^89^. In SH-SY5Y cells, Rab32 expression is elevated upon induction of ER stress (thapsigargin treatment), leading to mitochondrial dysfunction and neuronal death^90^. Furthermore, the ER chaperone GRP78 binds to IP3R1 during the ER stress response, releasing Ca^2+^ for influx into mitochondria and inducing cell death due to mitochondrial dysfunction^91^.

**Table 2:** MAM complexes related to ER stress.

Complex Name	Cells	Functional of mechanisms
IP3R1-GRP75-VDAC complex	pLE and pTr	Induces apoptosis through mitochondrial dysfunction and ER stress via the IP3R/GRP75/VDAC1-MCU axis
	ES2 and OV90	Induces ER stress with activation of the UPR due to increases in cytosolic and mitochondrial calcium
	BMECs	Induces ER stress and mitochondrial oxidative damage via the IP3R/GRP75/VDAC1-MCU axis
VAPB-PTPIP51 complex	NSC34	Induces inhibition of IRE1/XBP1 due to VAPB loss under ER stress conditions
	HEK293 and CV1	Induces Ca^2+^ regulation due to an interaction with PTPIP51 via VAPB-induced ER stress
MFN2-MFN2 complex	ES2 and OV90	Induces inhibition of cell growth under ER and mitochondrial stress conditions
Sig-1R	HEK293	Induces apoptosis via upregulation of the PERK/eIF2α/ATF4 pathway under ER stress conditions
Rab32	SH-SY5Y	Induces mitochondrial dysfunction and cell death with upregulation of GRP75 and MFN2 under ER stress conditions

Further evidence has also revealed that several MAM proteins affect UPR pathways. The ER protein VAPB is an important protein involved in UPR activity, and VABP loss inhibits IRE1/XBP1 activity in response to ER stress^92^. Furthermore, VAPB interacts with ATF6 in response to ER stress, and the terminal domain of ATF6 senses protein accumulation in the ER lumen. VAPB, with no luminal structure, is not directly regulated by ATF6 activation but is indirectly inhibited^93^. VAPB-induced ER stress has been implicated in inducing mitochondrial dysfunction by releasing Ca^2+^ through interactions with PTPIP51 in the mitochondrial membrane^23^.

## Characteristics and diagnostic role of ER–mitochondria contact sites in cancers

**Fig. 3:**
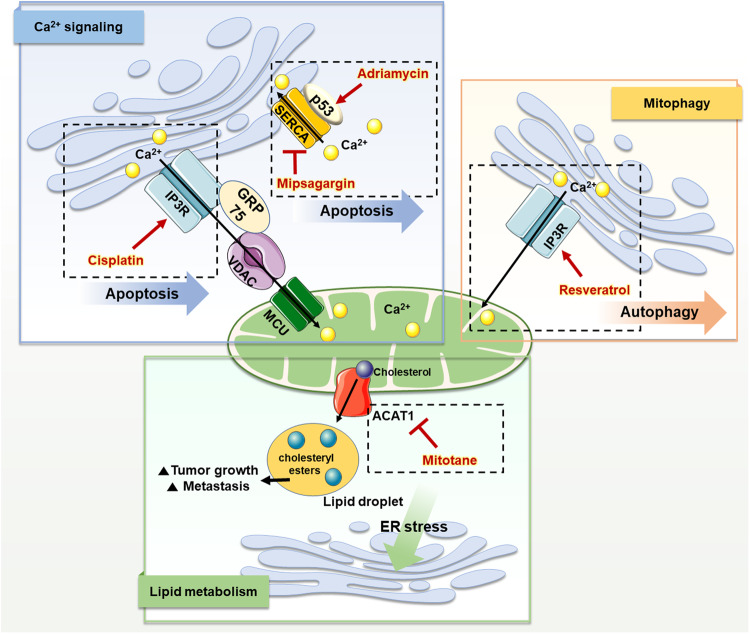
Representative characteristics of mitochondria-associated ER membranes (MAMs) in cancer and their therapeutic targets. The figure shows representative alterations in MAMs in cancer cells from three perspectives (Ca^2+^ signaling, mitophagy, and lipid metabolism) and the therapeutic drugs that target them. In cancer, the function of the IP3R-GRP75-VDAC complex is impaired, thus limiting Ca^2+^ trafficking to mitochondria and inducing resistance to mitochondrial apoptosis. Cisplatin targets IP3R and promotes the activity of its complex, which activates the influx of Ca^2+^ into mitochondria and induces apoptosis. Additionally, p53 mutations have been detected in various cancers, and these mutations result in the inhibition of Ca^2+^ influx into the ER and thus in cell death. Adriamycin increases p53 levels in MAMs and facilitates Ca^2+^ influx into the ER through SERCA, promoting apoptosis in cancer. Mipsagargin inhibits SERCA activity and increases the intracellular Ca^2+^ level, which can trigger cancer cell death. Resveratrol promotes Ca^2+^ signaling through IP3R, resulting in autophagy-induced cancer cell death. ACAT-1 generates cholesteryl esters that induce the accumulation of lipid droplets, resulting in tumor growth and metastasis. Mitotane inhibits ACAT-1 and causes free cholesterol accumulation in the ER, leading to ER stress-mediated apoptosis in cancer cells.

Cancer cells require a substantial amount of energy for their rapid proliferation and acquisition of malignant phenotypes and use various methods, such as increases in glucose uptake and glycolytic activity (a phenomenon known as the Warburg effect), lipid synthesis and lipolysis, and modulation of Ca^2+^ signaling, to meet these requirements^94–96^. Therefore, MAMs play important roles in cancer cell function and metabolism, as they regulate the aforementioned pathways (Fig. 3).

The regulation of Ca^2+^ signaling is crucial in cancers, as it is involved in cancer progression, epithelial-to-mesenchymal transition, invasion, and resistance to apoptosis^97^. Therefore, Ca^2+^-regulating proteins in MAMs play various roles in cancer development (Table 3). The IP3R-GRP75-VDAC-MCU complex, which plays an important role in Ca^2+^ transport, is regulated by oncoproteins such as PTEN, BRCA1, and BCL2^98^. In MAMs, PTEN binds to IP3R and prevents its degradation, thereby promoting Ca^2+^ transport to mitochondria, which is important for apoptosis^98,99^. However, in various cancers, PTEN loses functionality and triggers inappropriate Ca^2+^ transport, leading to apoptosis resistance^100,101^. BCL2, another oncoprotein in MAMs, interacts with IP3R and VDAC and prevents the translocation of Ca^2+^ from the ER to mitochondria. Furthermore, the interaction between BCL2 and VDAC1 interferes with the export of cytochrome c from mitochondria and thus hinders apoptosis^98,102^. Therefore, BCL2 overexpression in cancers results in resistance to apoptosis.

**Table 3:** Therapeutic strategies targeting MAM proteins in cancers.

Treatment approach	Target protein	Function	Therapeutic	Cancer
Inducing apoptosis through Ca^2+^ signaling	IP3R	Increase IP3R-mediated Ca^2+^ influx into mitochondria	Cisplatin	-Ovarian cancer
	P53	Increase p53 signaling related to SERCA activity and increase Ca^2+^ transfer to mitochondria, inducing apoptosis	Adriamycin	Almost all cancers
	ATP synthase	Inhibit ATP synthase, leading to SERCA activity and resulting in mitochondrial apoptosis	Resveratrol	Colorectal cancers
	SERCA	Inhibit SERCA, which increases intracellular Ca^2+^, inducing apoptosis	Mipsagargin	Prostate, breast and bladder cancers
	BCL2	Disrupt the interaction between BCL2 and IP3R, inducing Ca^2+^ release and apoptosis	BIRD2	Various cancers including large B-cell lymphoma, chronic lymphocytic leukemia
Inducing apoptosis through lipid metabolism	GRP78	Inhibit GRP78 and induce ER stress in cancer cells	HA-15	Human adrenocortical H295R cells
	ACAT-1	Inhibit ACAT-1, causing the accumulation of free cholesterol and fatty acids, thus inducing ER stress	Mitotane	Adrenocortical carcinoma
Increasing sensitivity to anticancer	GRP75	Knock down GRP75, increasing cisplatin sensitivity	-	Ovarian cancer
	BCL2	Inhibit BCL2, increasing intracellular Ca^2+^ levels	ABT737	Ovarian cancer
Reducing metastasis	FUNDC1	Inhibit FUNDC1, which regulates mitophagy and stimulates the proliferation and invasion of cancer cells	-	Breast cancer
	MCU	Inhibit MCU, leading to ROS suppression and HIF-1α downregulation, thus resulting in inhibition of tumor progression and metastasis	-	Breast, ovarian, prostate, and colorectal cancer
Increasing immune cell accessibility	-	Change glycan expression by modulating ER–mitochondria interactions	-	Glioblastoma

BRCA1-associated protein 1 (BAP1), a tumor suppressor protein in MAMs, facilitates Ca^2+^ influx into mitochondria by interacting with IP3R^103^. Abnormalities in the function of BAP1 can induce inappropriate Ca^2+^ influx into mitochondria, which may affect the regulation of apoptosis and lead to carcinogenesis^104^. Mutations in BAP1 have been observed in various cancers, including renal cell carcinoma, cutaneous melanoma, and uveal melanoma^104^. GRP75 also plays an important role in the regulation of Ca^2+^ homeostasis^2^. Transglutaminase type 2 modulates GRP75 function by binding to GRP75 and increasing Ca^2+^ flux between the ER and mitochondria, which affects cancer growth and metastasis. Upregulation of transglutaminase type 2 is a hallmark of breast cancer^105,106^. Additionally, TOM70, a protein that links IP3R3 to VDAC, exhibits notably high expression levels in breast cancer cells, and its potential as a therapeutic target has been duly recognized in previous studies^48,107^.

In addition to its proapoptotic role in mitochondria, Ca^2+^ is important for energy production, progression, and metastasis in cancer^108,109^. Ca^2+^ influx into mitochondria mediated by MCU promotes mitochondrial biogenesis and colon cancer proliferation^108^, and impairment of Ca^2+^ uptake by MCU knockdown inhibits the proliferation of embryonal rhabdomyosarcoma^110^. Other types of cancers with high MCU expression include prostate, ovarian, and breast cancers, indicating the diagnostic utility of MCU expression in cancer^111^. Moreover, PDZD8, another Ca^2+^-regulating protein in MAMs, was found to exhibit increased expression levels in stomach cancer tissue compared with normal tissue and is involved in the proliferation and metastasis of stomach cancer^112^.

Although research on the exact role of ROS in cancers is still underway, ROS are known to be involved in cancer progression and metastasis^98^. Several MAM proteins, including p66hsc, are regulated by ROS, and p66hsc and the oncoprotein p53 regulate each other^113^. Furthermore, p66hsc can be activated by steroid hormones, and activated p66hsc interacts with cytochrome c to increase ROS production. These alterations, including oxidative stress, have been reported to result in poor prognosis in patients with prostate cancer^72,114–116^. These characteristics of p66hsc have also been observed in other cancers, including breast and lung cancers, indicating its potential as a diagnostic and therapeutic target^117–119^. Furthermore, ERO1, which controls ROS production through the regulation of MAM proteins, is overexpressed in cholangiocarcinoma and is involved in proliferation and metastasis, leading to poor prognosis in patients^120^. Notably, ERO1 is also overexpressed in various other cancers, including breast cancer, lung cancer, and hepatocellular carcinoma, in which it ultimately results in poor prognosis^121–123^.

Activated lipid metabolism and the accumulation of lipid droplets are hallmarks of various cancer cells^95^. Elevated lipid levels in cancer cells promote proliferation and serve as energy reserves and messengers in oncogenic pathways^95,124^. Furthermore, various enzymes involved in lipid synthesis are upregulated in various cancers, including lung, ovarian, and prostate cancers^95,125,126^. Various enzymes involved in lipid synthesis, such as fatty acid CoA ligase, which catalyzes the ligation of triacylglycerols and ceramide, and acyl-coenzyme A:cholesterol acyltransferase-1 (ACAT-1), which catalyzes the synthesis of cholesterol, are mainly located in MAMs^98,127,128^. Therefore, alterations in the expression of these enzymes in MAMs are strongly associated with cancer. For example, after passing through mitochondria, ceramide plays an important role as an apoptosis inducer and can inhibit cancer growth and cell death^129,130^. Cholesterol metabolism is strongly associated with cancer. ACAT-1 in MAMs converts cholesterol to cholesteryl esters, which accumulate in the lipid droplets of cancer cells^98,131^. These accumulated cholesteryl esters have a considerable impact on the proliferation and metastasis of cancer cells^132^. Caveolin-1, located in MAMs, is involved in cholesterol efflux, and its overexpression has been identified in a variety of cancers, such as lung, liver, kidney, and colon cancers^133^. These expression patterns of caveolin-1 are closely related to cancer progression, metastasis, and drug resistance^134–136^. Therefore, various MAM proteins play major roles in cancer and can potentially be used in diagnosis and treatment. The association between ER stress and cancer has been established^137^. Sig-1R, a MAM protein regulated by ER stress, has been reported to be overexpressed in myelogenous leukemia and colon cancer^138^. This increased expression promotes angiogenesis and facilitates cancer cell migration, resulting in poor prognosis in patients. Consequently, Sig-1R is considered a promising therapeutic target^138^. Another MAM protein associated with ER stress, VAP-B, has been reported to play a key role in breast cancer progression, highlighting its potential as a diagnostic marker for this malignancy^139^.

## The ER–mitochondrial axis as a therapeutic target

### Targeting Ca^2+^ signaling

The characteristic functions of MAMs, including those in Ca^2+^ and ROS signaling, lipid metabolism, autophagy, and mitochondrial fission, enable their use as diagnostic markers and therapeutic targets for cancer (Fig. 3). Different methods can be used to trigger cancer cell apoptosis by promoting Ca^2+^ transport through modulation of MAM proteins. One of the most widely used anticancer drugs, cisplatin, is used to treat various cancers, including ovarian, breast, lung, and bladder cancers^140^. In ovarian cancer (SKOV3) cells, cisplatin promotes Ca^2+^ translocation from the ER to mitochondria and cytosol, causing ER stress-mediated apoptosis^141^. Other cancer therapeutics, such as adriamycin and mipsagargin, target Ca^2+^ signaling. In MAMs, p53 regulates the activity of SERCA by binding to it, leading to Ca^2+^ influx into the ER and resulting in increased apoptosis^142^. p53 mutations have been detected in various types of cancers, and adriamycin can increase p53 levels in MAMs, which promotes Ca^2+^ signaling and apoptosis in cancer cells through the activation of SERCA^111,142,143^. Mipsagargin inhibits SERCA function, resulting in an increase in intracellular Ca^2+^, which induces apoptosis in cancer cells^144^. Another component of the Ca^2+^ transport complex, VDAC, can potentially serve as a biomarker and therapeutic target for breast cancer, as its overexpression was detected in a previous study^145^. Furthermore, VDAC1 inhibition by siRNA induces cancer cell apoptosis, suggesting that siRNAs could be a target for cancer therapy^146,147^. Previous studies have shown that PDZD8, which is highly expressed in stomach cancer and is involved in cancer cell proliferation and metastasis, can also be used as a therapeutic target^112^. Notably, sunitinib, a kinase inhibitor, attenuates the proliferation of stomach cancer cells, as demonstrated in the human gastric cancer cell lines TMK1 and MKN74, by decreasing the PDZD8 protein level^112^.

### Targeting lipid metabolism and ER stress

Targeting the lipid metabolism-related functions of MAMs could aid in cancer treatment. For example, mitotane, which targets ACAT-1, converts cholesterol to CE and causes lipid droplet formation in various cancers^148^. In adrenocortical carcinoma, mitotane-induced ACAT-1 suppression induces free cholesterol and fatty acid accumulation in the ER, leading to apoptosis^111,148,149^. Modulation of ER stress also constitutes a potential therapeutic approach for cancer. In prostate cancer, corosolic acid modulates IRE1 and PERK signaling and induces ER stress, which promotes apoptosis and inhibits cell proliferation^150^. In hepatocarcinoma, 20(S)-protopanaxadiol can increase UPR activity and enhance the ER stress response by phosphorylating components of the PERK cascade, subsequently leading to increases in the expression of associated genes^151^. Moreover, previous studies have shown that panaxydol induces Ca^2+^ release from the ER through IP3R and activates the JNK pathway, causing ER stress, which is mediated by PERK^152,153^. These effects trigger apoptosis in renal carcinoma and prostate cancer cells^152,153^. Evodiamine is another therapeutic candidate that affects the JNK and PERK pathways. By modulating both pathways, evodiamine can induce apoptosis in ovarian cancer cells and reduce the extent of metastasis in colon cancer^153–155^.

### Increasing the sensitivity of cancer cells to chemotherapeutic compounds

Repeated use of chemotherapeutic drugs can result in resistance to them; therefore, other MAM proteins can be targeted to reduce this resistance. For example, cisplatin is widely used to treat ovarian cancer; however, its long-term use can induce cisplatin resistance in ovarian cancer cells, which is highly correlated with GRP75^156^. GRP75 knockdown increases cisplatin-induced apoptosis in ovarian cancer cells, suggesting a decrease in resistance^157,158^. Blocking the function of MAM-localized BCL2, which interacts with IP3R, via a BCL2 inhibitor disrupts Ca^2+^ translocation and leads to an increase in the cellular Ca^2+^ level in cisplatin-resistant ovarian cancer cells. Furthermore, a study demonstrated that ABT737, a BCL2 inhibitor, reduces the cisplatin resistance of SKOV3 ovarian cancer cells by modulating Ca^2+^ signaling^159,160^. These changes in cellular Ca^2+^ signaling lead to cisplatin-induced apoptosis, indicating that the regulation of MAM proteins could lower resistance to anticancer agents^159,160^. Targeting MAM-localized PERK is also a potential approach for treating resistant forms of cancers. PERK regulates ER stress, ROS production, and Ca^2+^ levels, all of which affect the apoptotic process^59,161^. Previous studies have reported that modulating PERK can induce apoptosis in endocrine-resistant breast cancer cells^161–163^. Another example of increased anticancer treatment sensitivity is that occurring after combination treatment with bortezomib, a proteasome inhibitor, and cisplatin. In pancreatic cancer, bortezomib can maximize the anticancer effects of cisplatin by activating JNK cascades and subsequently inducing apoptosis^159,164^.

### Reducing cancer metastasis

Emerging evidence suggests that MAM proteins and mitochondrial calcium dynamics may affect the migratory ability of cells^165^, and several studies have revealed the roles of MAM proteins in tumor invasion and metastasis, offering valuable perspectives for both diagnostic and therapeutic approaches. In triple-negative breast cancer cells, blocking MCU function can inhibit Ca^2+^ influx into mitochondria and ROS generation, resulting in reduced migration and progression^159,166^. Moreover, overexpression of FUN14 domain-containing 1 (FUNDC1), a MAM protein, could be a diagnostic and prognostic marker for breast cancer, as it triggers cell proliferation, migration, and invasion^167^. FUNDC1 knockdown by siRNA alters NFATC1 activity and inhibits the proliferation and metastasis of breast cancer cells^147,168^. A similar example is the targeting of MFN2 by miR-761 in hepatocellular carcinoma discovered in a previous study^165^, wherein miR-761 was shown to be upregulated in the tissues of patients with hepatocellular carcinoma, thereby confirming its role in modulating MFN2 expression. Additionally, miR-761 inhibition resulted in reduced migration and invasion of human cancer cell lines, as well as suppression of tumor metastasis in nude mice^165^.

### Increasing immune cell activity

Modulation of MAMs may aid in cancer treatment by increasing the accessibility of immune cells. For example, interactions between the ER and mitochondria regulate the expression of glycans, which can reduce immune cell accessibility in glioblastoma^169^. A previous study proposed the modification of glycan expression in glioblastoma through modulation of ER–mitochondria contact sites to enhance immune cell recognition as a potential approach for glioblastoma treatment^169^. Furthermore, regulation of MAM proteins in immune and cancer cells can aid in treatment. In memory T cells, promoting AKT signaling can inhibit the expression of MAM-localized GSK3b and strengthen the interaction between VDAC and HK-1, resulting in increased cellular respiration and functional acquisition^170^. These alterations play a significant role in the differentiation of memory T cells into effector T cells^170^. These studies suggest that the modulation of MAM proteins to increase immune cell activity offers various therapeutic benefits.

## Conclusion

Interactions between organelles are involved in many cellular functions. This review focuses specifically on the contact sites between the ER and mitochondria, known as MAMs. Various MAM proteins play important roles in the regulation of Ca^2+^ signaling, lipid metabolism, mitochondrial dynamics, oxidative stress, and ER stress. Therefore, alterations in MAM proteins can lead to changes in these mechanisms, resulting in the inhibition of apoptosis and increased resistance to anticancer drugs. Several therapeutic agents targeting MAM proteins have been reported to induce apoptosis and reduce antibiotic resistance and metastasis in cancer cells by modulating Ca^2+^ signaling and lipid metabolism. Owing to these diverse effects in cancers, research on MAM-targeting therapeutics should be ongoing. Moreover, as alterations in MAM proteins are characteristic of various cancers, they can potentially serve as diagnostic markers and therapeutic targets; however, further research is needed to determine whether they can be used as accurate biomarkers for specific cancers.
